# Association of acute kidney disease with the prognosis of ischemic stroke in the Third China National Stroke Registry

**DOI:** 10.1186/s12882-022-02817-4

**Published:** 2022-05-18

**Authors:** Yilun Zhou, Dongxue Wang, Hao Li, Yuesong Pan, Xianglong Xiang, Yu Wu, Xuewei Xie, Xianwei Wang, Yang Luo, Xia Meng, Jinxi Lin, Hong Wang, Yong Huo, Kunihiro Matsushita, Jing Chen, Fan Fan Hou, Yongjun Wang

**Affiliations:** 1 Department of Nephrology, Beijing Tiantan Hospital, Capital Medical University, Beijing, China; 2 China National Clinical Research Center for Neurological Diseases, Beijing, China; 3 Department of Neurology, Beijing Tiantan Hospital, Capital Medical University, Beijing, China; 4 Center of Stroke, Beijing Institute for Brain Disorders, Beijing, China; 5 Beijing Key Laboratory of Translational Medicine for Cerebrovascular Disease, Beijing, China; 6 Center for Metabolic Disease Research, Lewis Kats School of Medicine, Temple University, Philadelphia, USA; 7 Department of Nephrology, Beijing Shijitan Hospital, Capital Medical University, Beijing, China; 8 Department of Cardiology, Peking University First Hospital, Beijing, China; 9 Johns Hopkins Bloomberg School of Public Health, Baltimore, MD USA; 10 Department of Epidemiology, School of Public Health and Tropical Medicine, Tulane University, New Orleans, LA USA; 11 Department of Medicine, Tulane University School of Medicine, New Orleans, LA USA; 12 National Clinical Research Center for Kidney Disease, Nanfang Hospital, Southern Medical University, Guangzhou, China; 13 State Key Laboratory of Organ Failure Research, Nanfang Hospital, Southern Medical University, Guangzhou, China; 14 Guangzhou Regenerative Medicine and Health Guangdong Laboratory, Guangzhou, China; 15 Division of Nephrology, Nanfang Hospital, Southern Medical University, Guangzhou, China

**Keywords:** Acute kidney disease, Stroke, Incidence, Prognosis, Mortality

## Abstract

**Background:**

Acute kidney disease (AKD) evolves a spectrum of acute and subacute kidney disease requiring a global strategy to address. The present study aimed to explore the impact of AKD on the prognosis of ischemic stroke.

**Methods:**

The Third China National Stroke Registry (CNSR-III) was a nationwide registry of ischemic stroke or transient ischemic attack between August 2015 and March 2018. As a subgroup of CNSR-III, the patients who had serum creatinine (sCr) and serum cystatin C (sCysC) centrally tested on admission and at 3-month, and with 1-year follow-up data were enrolled. Modified AKD criteria were applied to identify patients with AKD during the first 3 months post stroke according to the guidelines developed by the Kidney Disease: Improving Global Outcomes in 2012. The primary clinical outcome was 1-year all-cause death, and secondary outcomes were stroke recurrence and post stroke disability.

**Results:**

Five thousand sixty-five patients were recruited in the study. AKD was identified in 3.9%, 6.7%, 9.9% and 6.2% of the patients by using sCr, sCr-based estimated glomerular filtration rate (eGFR_sCr_), sCysC-based eGFR (eGFR_sCysC_), and combined sCr and sCysC-based eGFR (eGFR_sCr+sCysC_) criteria, respectively. AKD defined as sCr or eGFR_sCr_ criteria significantly increased the risk of all-cause mortality (adjusted HR 2.67, 95% CI: 1.27–5.61; adjusted HR 2.19, 95% CI: 1.17–4.10) and post stroke disability (adjusted OR 1.60, 95% CI: 1.04–2.44; adjusted OR 1.51, 95% CI: 1.08–2.11). AKD diagnosed by eGFR_sCysC_ or eGFR_sCr+sCysC_ criteria had no significant impact on the risk of all-cause death and post stroke disability. AKD, defined by whichever criteria, was not associated with the risk of stroke recurrence in the adjusted model.

**Conclusions:**

AKD, diagnosed by sCr or eGFR_sCr_ criteria, were independently associated with 1-year all-cause death and post stroke disability in Chinese ischemic stroke patients.

**Supplementary Information:**

The online version contains supplementary material available at 10.1186/s12882-022-02817-4.

## Background

Acute kidney disease (AKD) was first proposed by the Kidney Disease: Improving Global Outcomes (KDIGO) Clinical Practice Guideline for Acute Kidney Injury (AKI) in 2012 [1]. The definition of AKD includes AKI, or a glomerular filtration rate (GFR) < 60 mL/min/1.73m^2^ for < 3 months, or a decrease in GFR by ≥ 35% for < 3 months, or an increase in serum creatinine (sCr) by > 50% for < 3 months, or any kidney damage lasting < 3 months [1]. The conceptual frame work of AKD is intended to cover the entire spectrum of acute and subacute stages which might not fulfill the strict criteria for AKI or chronic kidney disease (CKD), but still requires medical attention to prevent adverse outcomes. Up to now, limited clinical studies have characterized AKD [2, 3]. Accumulated evidence demonstrates that stroke patients with AKI or CKD would have higher risk of mortality, stroke recurrence and disability, but less is known about the impact of AKD on the prognosis of ischemic stroke [4–7].

The Third China National Stroke Registry (CNSR-III) was a cohort study in patients with acute ischemic stroke or transient ischemic attack (TIA) [8]. Two renal function markers, sCr and serum cystatin C (sCysC), were centrally tested on admission and at 3-month follow-up. Therefore, valuable data were available for initial estimation of a specific type of AKD, which focused on the changes in kidney function during the 3-month after stroke attack. We attempted to show whether stroke patients who developed AKD would be at higher risk of subsequent worse stroke outcomes.

## Methods

CNSR-III was a national, hospital-based, prospective study between August 2015 and March 2018, designed to evaluate the aetiology, imaging and biological markers for the prognosis of ischemic stroke or TIA. Patients older than 18 years old, and within 7 days from the onset of symptoms were enrolled. Special personnel were assigned to control the quality of data during the implementation of the project. Data clean was also conducted. Details of the CNSR-III cohort were described elsewhere [8]. In this subgroup study of CNSR-III, the population included those with sCr and sCysC centrally tested on admission and at 3-month after admission, and with 1-year follow-up data from CNSR-III. Patients’ baseline information was collected within 24 h after admission through a face-to-face interview by research coordinators. Demographic information included age, gender, body mass index (BMI) calculated as weight/height (kg/m^2^). Medical history included a history of diabetes, hypertension, dyslipidemia, and coronary heart disease. Patients were classified into different subtypes according to the TOAST criteria (Trial of Org 10,172 in Acute Stroke Treatment): large artery atherosclerosis, cardioembolism, small artery occlusion, other determined etiology and undetermined etiology [9]. Stroke severity was assessed by the National Institute of Health Stroke Scale (NIHSS) and disability was assessed by the modified Rankin Scale (mRS) within 24 h after admission [8]. The medications used during hospitalization, including dehydrant and angiotensin converting enzyme inhibitors/angiotensin receptor blockers (ACEI/ARBs), were collected.

In the present study, serum biomarkers for renal function evaluation included sCr and sCysC, and there were two time points for evaluating renal function. The blood samples were collected on the first day of enrolment and at 3-month, and stored in cryotube at − 80 °C refrigerator at clinical sites. The samples were transported through cold chain to the central laboratory in Beijing Tiantan Hospital, where all serum specimens were stored at − 80 °C until testing was performed. The value of sCr was measured by enzymatic method (sarcosine oxidase-PAP) using a commercial kit (Beckman Coulter, Brea, CA, USA) according to the manufacturer’s protocol. The value of sCysC was measured by the immunoturbidimetric method (Roche cobas c501 analyzer with Cystatin C assay), which had an approximate coefficient of variation of 2%. Study technicians running the assays were blinded to the participant’s clinical information. In order to capture more people who truly had AKD but did not have renal function data before the stroke attack, modified KDIGO AKD criteria was applied using a similar methodology to AKI studies [1, 10, 11]. (1) AKD defined by sCr: sCr measured at 3-month increases or decreases by > 50% of the value on admission; (2) AKD defined by estimated GFR (eGFR): eGFR measured at 3-month increases or decreases by ≥ 35% of the value on admission. eGFR were calculated as sCr-based eGFR (eGFR_sCr_), sCysC-based eGFR (eGFR_sCysC_), and combined sCr and sCysC-based eGFR (eGFR_sCr+sCysC_) using the equations from the Chronic Kidney Disease Epidemiology Collaboration (CKD-EPI) [12].

Primary outcome was all-cause death which occurred from 3-month to 1-year. Secondary outcomes included stroke recurrence defined as new ischemic stroke and haemorrhagic stroke from 3-month to 1-year, and post stroke disability defined by scores on the mRS range from 3 to 6 at 1-year.

The SAS 9.4 (SAS Institute Inc., Cary, NC) statistical analysis software was used for data processing. Continuous variables were expressed as mean with standard deviation (SD) or median with interquartile range (IQR); categorical variables were expressed as number with percentage. Continuous variables between groups were compared using t test, or Mann–Whitney U test; categorical variables were compared using χ^2^ test. Survival curves were estimated by the Kaplan–Meier method and compared by the log rank test. Cox proportional hazard regression model or multivariate logistic regression analysis was used for assessment of variables that were associated with all-cause death, stroke recurrence or stroke disability by calculating hazard ratios / odds ratio (HR/OR) and 95% confidence interval (CI). *P* value less than 0.05 was considered statistically significant. The proportional hazard assumption for the Cox regression model was examined by including a time-dependent covariate with interaction of AKD into the model. Multicollinearity analysis, and analysis of the interaction between AKD and variables in the Cox proportional hazard model were performed.

## Results

**Fig. 1:**
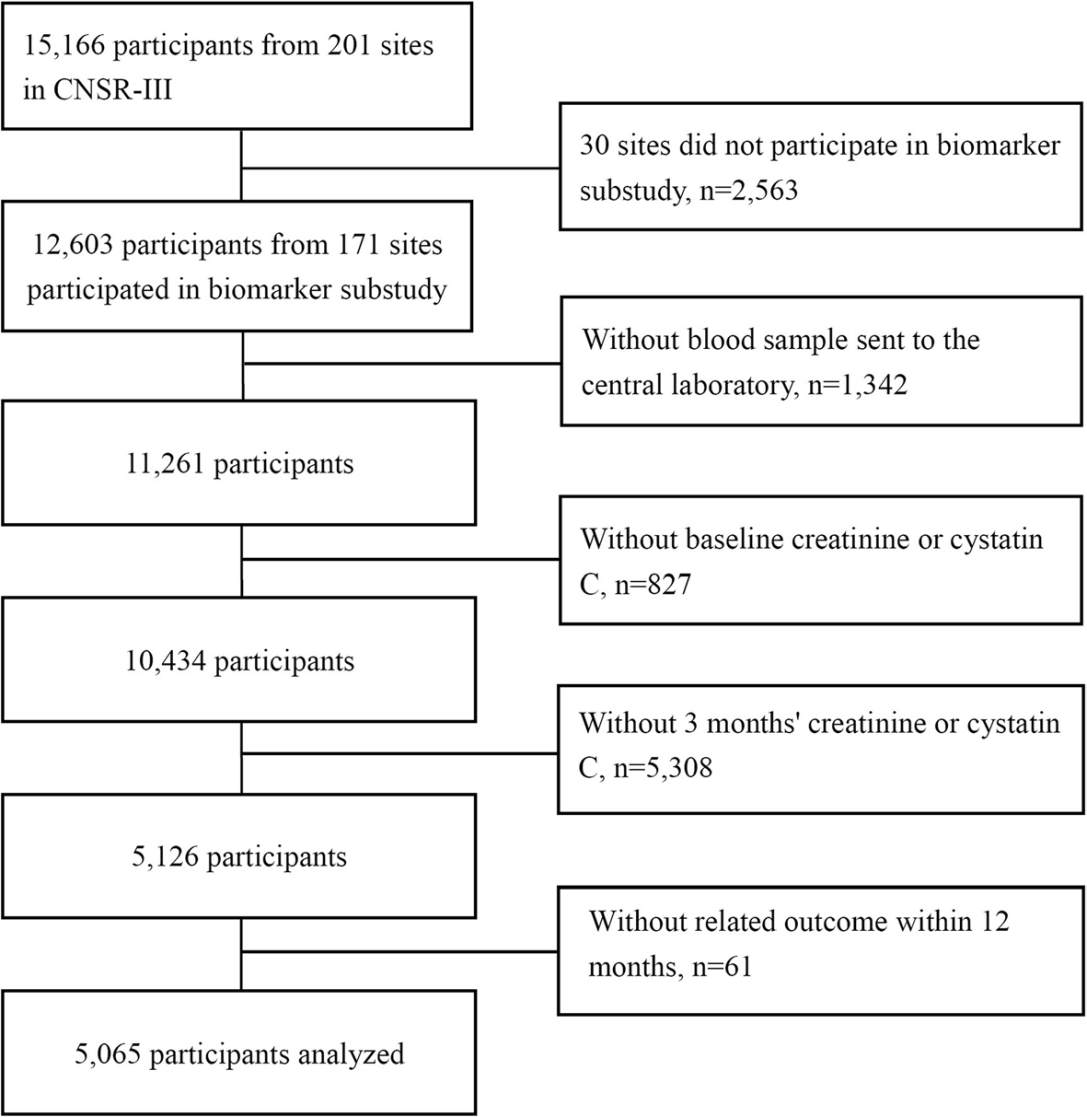
Study flow chart. CNSR-III, the Third China National Stroke Registry-III

Among 15,166 participants in the CNSR-III, 12,603 participants participated in biomarker substudy, and 11,261 participants were with blood sample sent to the central laboratory. There were 827 patients excluded due to without sCr or sCysC data on admission, and 5,308 patients were excluded without sCr or sCysC data at 3-month. 61 patients were lost to follow-up. Eventually, 5,065 patients were included in this study, shown in Fig. 1. The study population was from 171 hospitals in 25 provinces and 4 municipalities across China. The baseline clinical characteristics of patients on admission were presented in Table 1. Demographic data showed that 59.9% of patients were older than 60 years, 68.2% were male, and 67.9% had the NIHSS score less than 5 on admission. There was 6.4% of patients with eGFR_sCr_ < 60 mL/min/1.73m^2^, 14.6% with eGFR_sCysC_ < 60 mL/min/1.73m^2^, and 8.4% with eGFR_sCr+sCysC_ < 60 mL/min/1.73m^2^ on admission, respectively (Table 1). The median value of sCr was 69.0 (58.0–80.0) umol/L on admission, and 68.0 (58.0–80.0) umol/L at 3-month. The median value of sCysC was 0.94 (0.83–1.07) mg/L on admission, and 1.01 (0.89–1.18) mg/L at 3-month. sCr levels at 3-month decreased by 1.4% of the value on admission, and sCysC increased by 7.4%. Demographic data about excluded patients were shown in Supplemental Material (Table S1, available as online supplementary material).

**Table 1:** Characteristics for stroke patients with and without AKD

	**Total**	**sCr based AKD**			**eGFR**_**sCr**_** based AKD**			**eGFR**_**sCysC**_** based AKD**			**eGFR**_**sCr+sCysC**_** based AKD**		
		**Non-AKD**	**AKD**	**P**	**Non-AKD**	**AKD**	**P**	**Non-AKD**	**AKD**	**P**	**Non-AKD**	**AKD**	**P**
n (%)	5065(100.0)	4867(96.1)	198(3.9)	––	4728(93.3)	337(6.7)	––	4565(90.1)	500(9.9)	––	4751(93.8)	314(6.2)	––
Age (year), n (%)				0.83			0.008			0.01			0.34
< 60	2032(40.1)	1954(40.1)	78(39.4)		1920(40.6)	112(33.2)		1857(40.7)	175(35.0)		1914(40.3)	118(37.6)	
≥ 60	3033(59.9)	2913(59.9)	120(60.6)		2808(59.4)	225(66.8)		2708(59.3)	325(65.0)		2837(59.7)	196(62.4)	0.34
Male gender, n (%)	3453(68.2)	3327(68.4)	126(63.6)	0.16	3237(68.5)	216(64.1)	0.10	3107(68.1)	346(69.2)	0.60	3255(68.5)	198(63.1)	0.04
TIA, n (%)	412(8.1)	395(8.1)	17(8.6)	0.81	381(8.1)	31(9.2)	0.46	364(8.0)	48(9.6)	0.21	382(8.0)	30(9.6)	0.34
TOAST, n (%)				0.13			0.17			0.004			0.10
SAO	1213(24.0)	11,178(24.2)	35(17.7)		1149(24.3)	64(19.0)		1118(24.5)	95(19.0)		1155(24.3)	58(18.5)	
LAA	1252(24.7)	1193(24.5)	59(29.8)		1163(24.6)	89(26.4)		1109(24.3)	143(28.6)		1165(24.5)	87(27.7)	
CE	265(5.2)	255(5.2)	10(5.1)		245(5.2)	20(5.9)		229(5.0)	36(7.2)		245(5.2)	20(6.4)	
Other or undetermined	2335(46.1)	2241(46.0)	94(47.5)		2171(45.9)	164(48.7)		2109(46.2)	226(45.2)		2186(46.0)	149(47.5)	
Medical history													
Hypertension	3177(62.7)	3045(62.6)	132(66.7)	0.24	2945(62.3)	232(68.8)	0.02	2816(61.7)	361(72.2)	< 0.001	2955(62.2)	222(70.7)	0.003
Diabetes	1158(22.9)	1097(22.5)	61(30.8)	0.007	1057(22.5)	101(30.0)	0.001	1019(22.3)	139(27.8)	0.006	1067(22.5)	91(29.0)	0.008
Dyslipidemia	437(8.6)	422(8.7)	15(7.6)	0.59	402(8.5)	35(10.4)	0.23	385(8.4)	52(10.4)	0.14	398(8.4)	39(12.4)	0.01
Coronary heart disease	527(10.4)	506(10.4)	21(10.6)	0.92	481(10.2)	46(13.7)	0.04	459(10.1)	68(13.6)	0.01	482(10.2)	45(14.3)	0.02
Clinical characteristics on admission													
NIHSS, n (%)				0.01			< 0.001			< 0.001			0.25
< 5	3441(67.9)	3325(68.3)	116(58.6)		3242(68.6)	199(58.1)		3146(68.9)	295(59.0)		3241(68.2)	200(63.7)	
5–15	1548(30.6)	1471(30.2)	77(38.9)		1423(30.1)	125(37.1)		1353(29.6)	195(39.0)		1439(30.3)	109(34.7)	
> 15	76(1.5)	71(1.5)	5(2.5)		63(1.3)	13(3.9)		66(1.5)	10(2.0)		71(1.5)	5(1.6)	
mRS, n (%)				0.08			0.07			< 0.001			0.04
0–2	3626(71.6)	3495(71.8)	131(66.2)		3399(71.9)	227(67.4)		3301(72.3)	325(65.0)		3417(71.9) 1334(28.08)	209(66.6)	
3–5	1439(28.4)	1372(28.2)	67(33.8)		1329(28.1)	110(32.6)		1264(27.7)	175(35.0)		1334(28.08)	105(33.4)	
BMI (Kg/m^2^), mean ± SD	24.8 ± 3.3	24.8 ± 3.3	24.4 ± 3.4	0.11	24.8 ± 3.3	24.6 ± 3.8	0.44	24.8 ± 3.3	24.5 ± 3.4	0.10	24.8 ± 3.3	24.5 ± 3.9	0.19
sCr (umol/L), median (IQR)	69.0(58.0–80.0)	69.0(59.0–80.0)	54.5(29.0–103.0)	< 0.001	68.0(58.0–79.0)	101.0(69.0–125.0)	< 0.001	69.0(58.0–80.0)	71.0(60.0–84.5)	< 0.001	69.0(58.0–80.0)	78.0(53.0–114.0)	< 0.001
sCysC (mg/L), median (IQR)	0.94(0.83–1.07)	0.94(0.83–1.07)	0.96(0.80–1.19)	0.11	0.93(0.83–1.06)	1.05(0.90–1.30)	< 0.001	0.93(0.83–1.06)	0.98(0.84–1.20)	< 0.001	0.93(0.83–1.06)	1.04(0.86–1.36)	< 0.001
eGFR_sCr_ (mL/min/1.73m^2^), n (%)				< 0.001			< 0.001			< 0.001			< 0.001
< 60	325(6.4)	272(5.6)	53(26.8)		168(3.6)	157(46.6)		266(5.8)	59(11.8)		220(4.6)	105(33.4)	
60 ~ 89	1649(32.6)	1626(33.4)	23(11.6)		1551(32.8)	98(29.1)		1462(32.0)	187(37.4)		1574(33.1)	75(23.9)	
≥ 90	3091(61.0)	2969(61.0)	122(61.6)		3009(63.6)	82(24.3)		2837(62.2)	254(50.8)		2957(62.2)	134(42.7)	
eGFR_sCysC_ (mL/min/1.73m^2^), n (%)				< 0.001			< 0.001			< 0.001			< 0.001
< 60	741(14.6)	690(14.2)	51(25.8)		627(13.3)	114(33.8)		599(13.1)	142(28.4)		626(13.2)	115(36.6)	
60 ~ 89	2427(47.9)	2347(48.2)	80(40.4)		2280(48.2)	147(43.6)		2224(48.7)	203(40.6)		2312(48.7)	115(36.6)	
≥ 90	1897(37.5)	1830(37.6)	67(33.8)		1821(38.5)	76(22.6)		1742(38.2)	155(31.0)		1813(38.2)	84(26.8)	
eGFR_sCr+sCysC_ (mL/min/1.73m^2^), n (%)				< 0.001			< 0.001			< 0.001			< 0.001
< 60	425(8.4)	371(7.6)	54(27.3)		293(6.2)	132(39.2)		344(7.5)	81(16.2)		320(6.7)	105(33.4)	
60 ~ 89	2266(44.7)	2227(45.8)	39(19.7)		2136(45.2)	130(38.6)		2036(44.6)	230(46.0)		2170(45.7)	96(30.6)	
≥ 90	2374(46.9)	2269(46.6)	105(53.0)		2299(48.6)	75(22.3)		2185(47.9)	189(37.8)		2261(47.6)	113(36.0)	
Medication in hospitalization, n (%)													
Dehydrant	186(3.7)	178(3.7)	8(4.0)	0.78	168(3.6)	18(5.3)	0.09	156(3.4)	30(6.0)	0.004	169(3.6)	17(5.4)	0.09
ACEI/ARBs	829(16.4)	794(16.3)	35(17.7)	0.61	770(16.3)	59(17.5)	0.56	725(15.9)	104(20.8)	0.005	772(16.3)	57(18.2)	0.38

*AKD* Acute kidney disease, *SD* Standard deviation, *IQR* Interquartile range, *sCr* Serum creatinine, *sCysC* Serum cystatin C, *eGFR* Estimated glomerular filtration rate, *TIA* Transient ischemic attack, *TOAST* Stroke subtype defined by the Trial of Org 10,172 in Acute Stroke Treatment classification, *LAA* Large-artery atherosclerosis, *SAO* Small-artery occlusion, *CE* Cardioembolism; mRS, modified Rankin Scale, *NIHSS* National Institutes of Health stroke scale, *BMI* Body mass index, *ACEI/ARBs* Angiotensin converting enzyme inhibitors/angiotensin receptor blockers

**Fig. 2:**
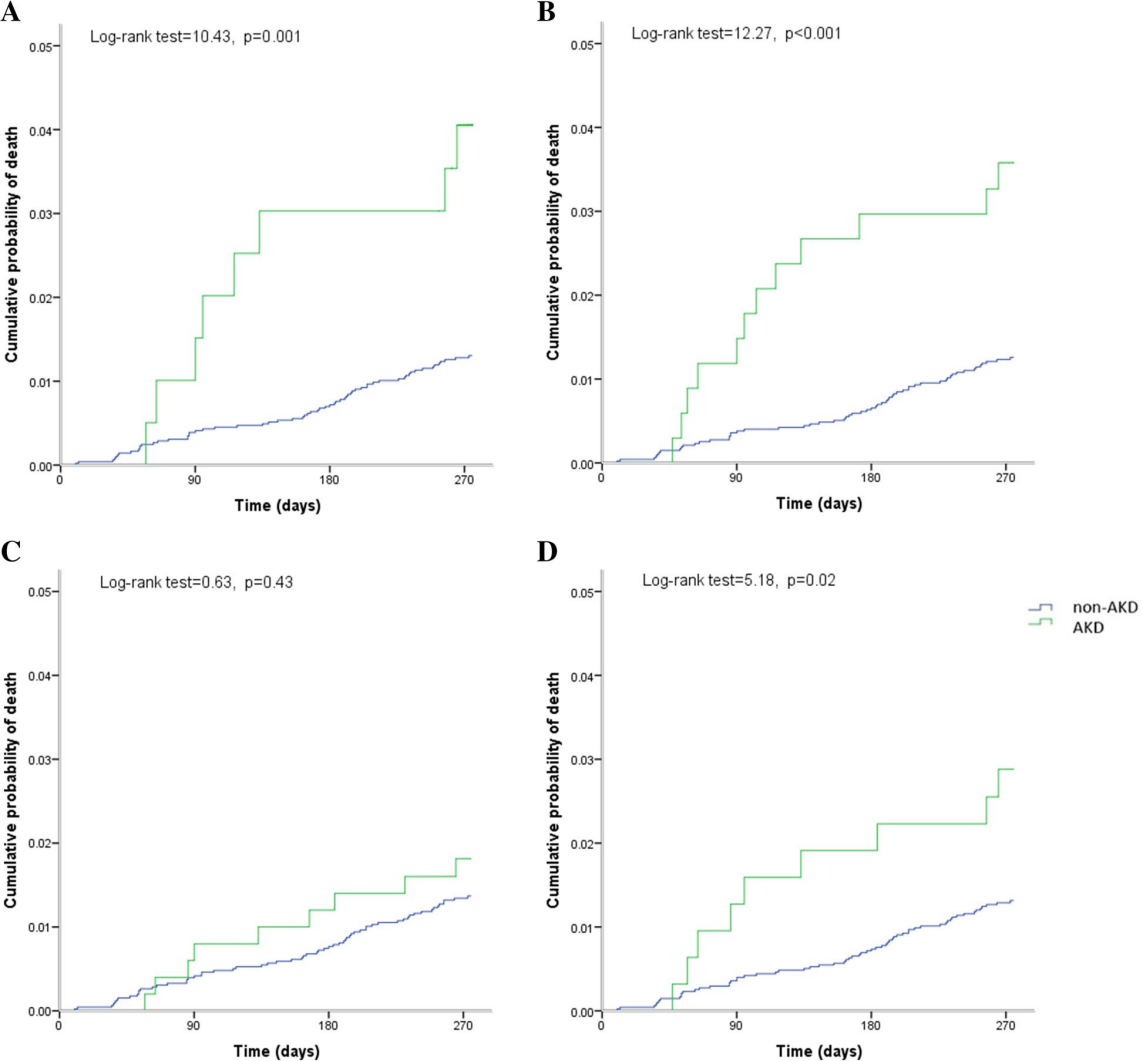
Kaplan–Meier estimates of probability of all-cause death by the development of AKD. (**A)** sCr-based criteria, (**B**) eGFR_sCr_-based criteria (**C**) eGFR_sCysC_-based criteria, and (**D**) eGFR_sCr+sCysC_-based criteria. X-coordinate from 3-month to 1-year. AKD, acute kidney disease; sCr, serum creatinine; sCysC, serum cystatin C; eGFR, estimated glomerular filtration rate

**Fig. 3:**
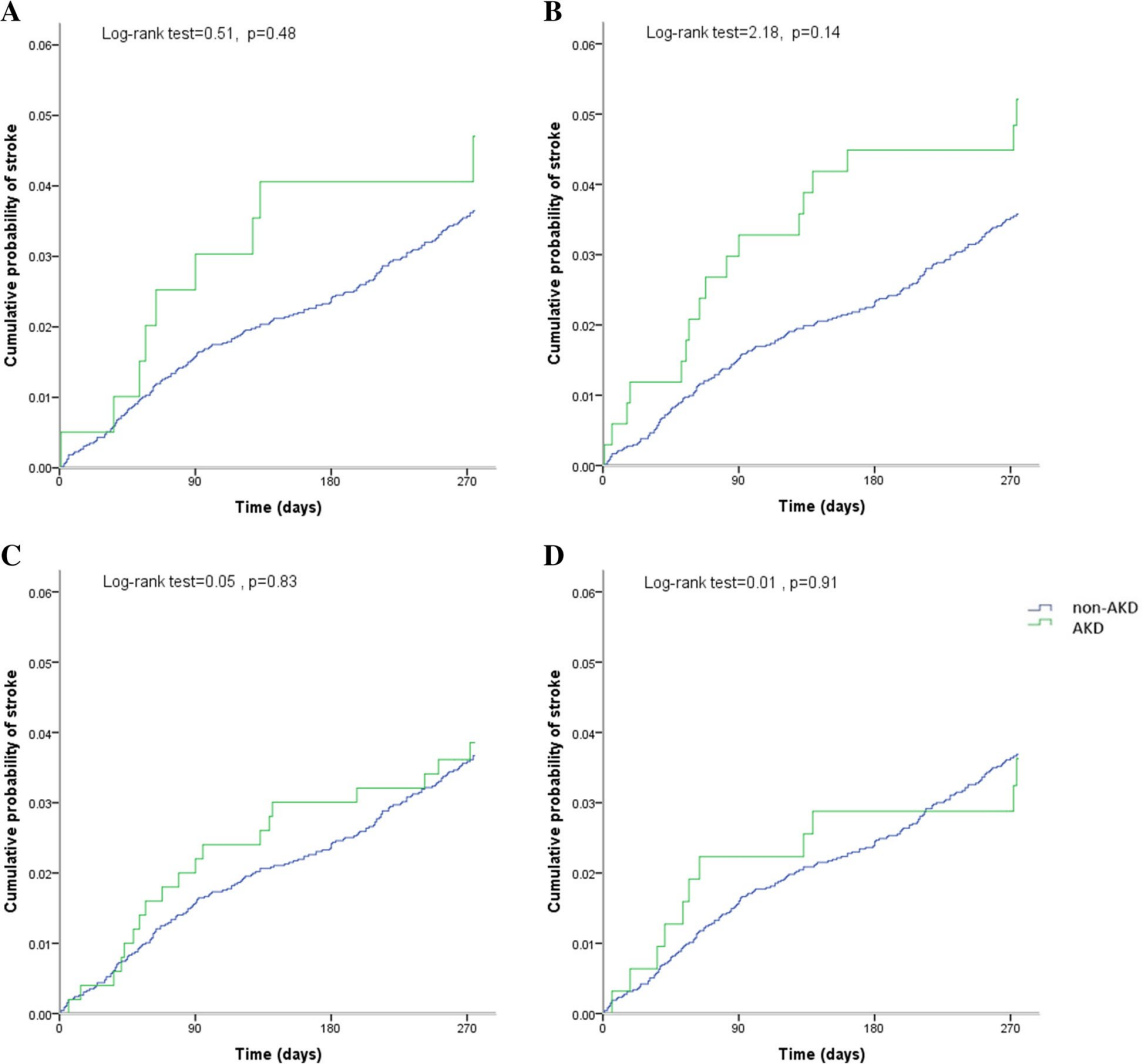
Kaplan–Meier estimates of probability of stroke recurrence by the development of AKD. (**A)** sCr-based criteria, (**B**) eGFR_sCr_-based criteria (**C**) eGFR_sCysC_-based criteria, and (**D**) eGFR_sCr+sCysC_-based criteria. X-coordinate from 3-month to 1-year. AKD, acute kidney disease; sCr, serum creatinine; sCysC, serum cystatin C; eGFR, estimated glomerular filtration rate

Of the 5,065 patients, AKD was diagnosed in 198 (3.9%), 337 (6.7%), 500 (9.9%) and 314 (6.2%) patients based on sCr, eGFR_sCr_, eGFR_sCysC,_ and eGFR_sCr+sCysC_, respectively (Table 1). AKD patients, compared to non-AKD patients, were more likely to have a history of diabetes and hypertension, had higher NIHSS score and higher percentage of eGFR < 60 mL/min/1.73m^2^ on admission (Table 1). Since 3 months after patient enrolment, 71 (1.4%) deaths, and 184 (3.6%) stroke recurrence, have occurred within the subsequent 9-month follow-up. The specific causes of death for the 71 patients were as follows: ischemic stroke, 7 patients; hemorrhagic stroke, 12 patients; acute myocardial infarction and other cardiovascular death, 7 patients; non-vascular death and undetermined cause, 45 patients. 491 (9.7%) post stroke disability occurred at 1-year. All of the proportional hazard assumptions were met (*P* value greater than 0.05), and the statistical analysis results were listed in Table S2 (available as online supplementary material). As shown in Table 2, AKD, defined as sCr or eGFR_sCr_-based criteria, significantly increased the risk of all-cause mortality even adjusted by the confounders. AKD diagnosed by eGFR_sCysC_ or eGFR_sCr+sCysC_-based criteria had no significant impact on the adjusted HR of primary outcome. Similarly, sCr or eGFR_sCr_-based, but not eGFR_sCysC_ or eGFR_sCr+sCysC_-based AKD, remarkably increased the risk of post stroke disability. Notably, AKD, no matter which criteria was used, was not associated with the risk of stroke recurrence in the adjusted model. No obvious collinearities between variables in Cox proportional hazard model were found (all variance inflation factors far less than 10), as shown in Table S3 (available as online supplementary material). The Kaplan–Meier estimates of probability of the primary and secondary outcomes were shown in Fig. 2 and Fig. 3.

**Table 2:** Prognostic analysis of AKD associated with 1-year primary and secondary outcomes

	**sCr based AKD**			**eGFR**_**sCr**_** based AKD**			**eGFR**_**sCysC**_** based AKD**			**eGFR**_**sCr+sCysC**_** based AKD**		
	**AKD** ***n* = 198**	**non-AKD** ***n* = 4867**	**P**	**AKD** ***n* = 337**	**non-AKD** ***n* = 4728**	**P**	**AKD** ***n* = 500**	**non-AKD** ***n* = 4565**	**P**	**AKD** ***n* = 314**	**non-AKD** ***n* = 4751**	**P**
**All-cause death**^**a**^**, n(%)**	8 (4.0)	63(1.3)		12(3.6)	59(1.3)		9(1.8)	62(1.4)		9(2.9)	62(1.3)	
Crude HR (95% CI)	3.15(1.51–6.58)	ref	0.002	2.88(1.55–5.36)	ref	< 0.001	1.33(0.66–2.67)	ref	0.43	2.21(1.10–4.44)	ref	0.03
Adjusted HR (95% CI)^d^	2.67(1.27–5.61)	ref	0.01	2.19(1.17–4.10)	ref	0.02	1.01(0.50–2.05)	ref	0.97	1.86(0.92–3.76)	ref	0.08
**Stroke recurrence**^**b**^**, n(%)**	9(4.6)	175(3.6)		17(5.0)	167(3.5)		19(3.8)	165(3.6)		11(3.5)	173(3.6)	
Crude HR (95% CI)	1.28(0.65–2.49)	ref	0.48	1.45(0.88–2.39)	ref	0.14	1.05(0.66–1.69)	ref	0.83	0.97(0.53–1.78)	ref	0.91
Adjusted HR (95% CI)^d^	1.22(0.62–2.39)	ref	0.56	1.35(0.82–2.23)	ref	0.25	0.94(0.58–1.52)	ref	0.80	0.91(0.49–1.68)	ref	0.76
**Stroke disability**^**c**^**, n(%)**	32(16.2)	459(9.4)		56(16.6)	435(9.2)		69(13.8)	422(9.2)		42(13.4)	449(9.5)	
Crude OR (95% CI)	1.85(1.25–2.74)	ref	0.002	1.97(1.45–2.66)	ref	< 0.001	1.57(1.20–2.07)	ref	0.001	1.48(1.05–2.08)	ref	0.02
Adjusted OR (95% CI)^d^	1.60(1.04–2.44)	ref	0.03	1.51(1.08–2.11)	ref	0.02	1.20(0.89–1.62)	ref	0.23	1.26(0.88–1.82)	ref	0.21

*AKD* Acute kidney disease, *sCr* Serum creatinine, *sCysC* Serum cystatin C, *eGFR* Estimated glomerular filtration rate, *CI* Confidence interval, *OR* Odds ratio, *HR* Hazard ratio, *mRS* Modified Rankin Scale, *NIHSS* National Institutes of Health stroke scale, *TOAST* Trial of Org 10,172 in Acute Stroke Treatment

^**a**^All-cause death, defined as all-cause death occurred from 3-month to 1-year

^**b**^Stroke recurrence defined as stroke recurrence occurred from 3-month to 1-year

^**c**^Stroke disability, defined as a mRS score of 3–6 at 1-year

^**d**^Adjusted factors in multivariable analysis: age, gender, TOAST subtypes, mRS and NIHSS on admission, history of diabetes, hypertension, coronary heart disease

## Discussion

This is the first study, to our knowledge, to investigate the impact of AKD on the clinical outcomes of ischemic stroke. The results showed that AKD, defined by sCr or eGFR_sCr_-based criteria, significantly increased the risk of all-cause death and post stroke disability during a 1-year follow-up after ischemic stroke.

AKI is an abrupt decrease in kidney function occurring over 7 days or less, whereas CKD is defined by the persistence of kidney disease for a period of ≥ 3 months. In 2012, the KDIGO AKI workgroup proposed the term AKD to define any acute kidney diseases and disorders lasting for a period of < 3 months, that encompasses both AKI and any newly recognized kidney disease that does not meet the current definitions for AKI or CKD [1]. In this study, we used similar methodology to AKI studies to modify AKD criteria developed by KDIGO in 2012, as increase or decrease in sCr > 50% or eGFR ≥ 35% of the admission level at 3 months post admission [1, 10, 11]. Previous studies have demonstrated the concomitant renal dysfunction in patients with stroke, either AKI or CKD, were associated with morbidity and mortality [4–7]. However, the impact of AKD on the clinical outcomes after stroke has not been investigated. In the study, the risk of all-cause mortality was 2.67 folds in those with AKD defined by sCr criteria compared to those without AKD. Development of AKD also increased the risk of post stroke disability (adjusted OR 1.60, 95% CI: 1.04–2.44), suggesting that AKD occurred within 3 months post stroke may negatively impact the outcome of stroke. Given that sCr is accessible and inexpensive, the present study supports to use sCr to detect AKD after stroke.

Cystatin C alone or in combination with creatinine was reported to strengthen the association between the eGFR and the risks of death and end-stage renal disease in general-population or CKD [13]. While in the present study, no association was observed between either eGFR-based AKD involving sCysC (eGFR_sCysC_ or eGFR_sCr+sCysC_) and the 1-year clinical outcomes. Cystatin C, as an inhibitor of cysteine proteases, plays an important role in the pathogenesis of atherosclerosis [14]. The sCysC concentrations was reported to be significantly higher in patients with acute ischemic stroke than in the control group, and sCysC was independently associated with acute ischemic stroke [15]. On the other hand, increased cystatin C was suggested to be involved in endogenous neuroprotection, and exogenous cystatin C was found to exerte neuroprotective effects by reducing infarct volume in the animal stroke model [16, 17]. In the present study, the sCysC values increased by 7.4% at 3-month compared with admission, in contrast to sCr which was relatively stable (down 1.4%). Whether sCysC up-regulation represents a neuroprotective compensatory response, warrants further investigation. We hypothesized that, factors associated with ischemic stroke, may influence sCysC levels at 3 months and therefore weaken the relationship between eGFR_sCysC_ or eGFR_sCr+sCysC_-based AKD and clinical outcomes.

There were some limitations. Firstly, two-thirds of patients were excluded due to without blood sample or missing sCr or sCysC data. The excluded patients were those with higher NIHSS scores on admission, thus the prevalence of AKD in stroke population may be underestimated. Secondly, as the CNSR-III was carried out in 2015, the updated AKD definition by the Acute Disease Quality Initiative 16 Workgroup in 2017 was not applied in the study [18], and AKI could not be defined due to the lack of the renal function data during 7 days. Thirdly, we had incomplete data on assessment of muscle mass and other clinical conditions that might affect sCr or sCysC levels independently of GFR. More in-depth evaluation in the clinical practice was warranted.

## Conclusions

In summary, AKD, defined by sCr or eGFR_sCr_ criteria, was independently associated with 1-year all-cause mortality and stroke-induced disability in patients with acute ischemia stroke. Monitoring the change in sCr on a regular basis after attack of stroke may help to identify patients with AKD who are at high risk of adverse outcomes of stroke.

## Supplementary Information

**Additional file 1.**

## Data Availability

Anonymized data are available to researchers on request for reproducing the results or replicating the procedures by contacting the corresponding author.

## Abbreviations

AKD: Acute kidney disease
CNSR-III: The Third China National Stroke Registry-III;
KDIGO: Kidney Disease Improving Global Outcomes
AKI: Acute Kidney Injury
CKD: Chronic kidney disease
sCr: Serum creatinine
sCysC: Serum cystatin C
eGFR: Estimated glomerular filtration rate
TIA: Transient ischemic attack
NIHSS: National Institutes of Health Stroke Scale
mRS: Modified Ranking Scale
BMI: Body mass index
TOAST: Trial of Org 10,172 in Acute Stroke Treatment
LAA: Large-artery atherosclerosis
SAO: Small-artery occlusion
CE: Cardioembolism
ACEI/ARBs: Angiotensin converting enzyme inhibitors/angiotensin receptor blockers
CKD-EPI: Chronic Kidney Disease Epidemiology Collaboration
OR: Odds ratios
HR: Hazard ratio
CI: Confidence intervals
SD: Standard deviation
IQR: Interquartile range
