# Supplementary material for: Association of acute kidney disease with the prognosis of ischemic stroke in the Third China National Stroke Registry
Source: BMC Nephrol. 2022 May 18;23:188. doi: 10.1186/s12882-022-02817-4 (PMC9115968; doi:10.1186/s12882-022-02817-4)
Supplement: Supplementary file 1 — Additional file 1. [file 12882_2022_2817_MOESM1_ESM.docx]

**Table S1. Comparisions between included patients and excluded patients**

|  | Included patients | Excluded patients | P |
| --- | --- | --- | --- |
| n(%) | 5065(33.4) | 10101(66.6) | ---- |
| Age (year), n (%)  <60  ≥60 | 2032(40.1)  3033(59.9) | 3856(38.2)  6245(61.8) | 0.02 |
| Male gender, n (%) | 3453(68.2) | 6911(68.4) | 0.76 |
| TIA, n (%) | 412(8.1) | 608(6.0) | <0.001 |
| TOAST, n (%)  SAO  LAA  CE  Undetermined | 1213(24.0)  1252(24.7)  265(5.2)  2335(46.1) | 1952(19.3)  2604(25.8)  652(6.5)  4893(48.4) | <0.001 |
| Medical history, n (%) |  |  |  |
| Hypertension | 3177(62.7) | 6317(62.5) | 0.82 |
| Diabetes | 1158(22.9) | 2352(23.3) | 0.56 |
| Dyslipidemia | 437(8.6) | 754(7.5) | 0.01 |
| Coronary heart disease | 527(10.4) | 1081(10.7) | 0.58 |
| Clinical characteristics on admission |  |  |  |
| NIHSS, n (%)  <5  5-15  >15 | 3441(67.9)  1548(30.6)  76(1.5) | 6474(64.1)  3358(33.2)  269(2.7) | <0.001 |
| mRS, n (%)  0-2  3-5 | 3626(71.6)  1439(28.4) | 6737(66.7)  3364(33.3) | <0.001 |
| BMI (Kg/m^2^), mean± SD | 24.8±3.3 | 24.7±3.3 | 0.15 |
| Medications in hospitalization, n (%) |  |  |  |
| Dehydrant | 186(3.7) | 601(6.0) | <0.001 |
| ACEI/ARBs | 829(16.4) | 1645(16.3) | 0.90 |

TIA: transient ischemic attack; TOAST: Trial of Org 10172 in Acute Stroke Treatment; LAA: large-artery atherosclerosis; SAO: small-artery occlusion; CE: cardioembolism; NIHSS: National Institutes of Health Stroke Scale; mRS: modified Ranking Scale; BMI: body mass index; ACEI/ARBs: angiotensin converting enzyme inhibitors / angiotensin receptor blockers; HR: hazard ratio; CI: confidence intervals; SD: standard deviation.

**Table S2. The assumption of the Cox proportional hazard models**

|  | sCr based AKD,  P **^a^** | eGFR_sCr_ based AKD,  P **^a^** | eGFR_sCysC_ based AKD,  P **^a^** | eGFR_sCr+sCysC_ based AKD,  P **^a^** |
| --- | --- | --- | --- | --- |
| All-cause death |  |  |  |  |
| Univariate analysis | 0.124 | 0.390 | 0.519 | 0.212 |
| Multivariate analysis | 0.285 | 0.868 | 0.425 | 0.367 |
| Stroke recurrence |  |  |  |  |
| Univariate analysis | 0.720 | 0.468 | 0.927 | 0.566 |
| Multivariate analysis | 0.793 | 0.606 | 0.599 | 0.817 |

**^a^** Accessed by including a time-dependent covariate with interaction of AKD into the model; AKD: acute kidney disease; sCr: serum creatinine; sCysC: serum cystatin C; eGFR: estimated glomerular filtration rate.

**Table S3. Multicollinearity analysis for variables inthe Cox proportional hazard model**

|  | Variance Inflation Factor | | | |
| --- | --- | --- | --- | --- |
|  | sCr based AKD | eGFR_sCr_ based AKD | eGFR_sCysC_ based AKD | eGFR_sCr+sCysC_ based AKD |
| AKD | 1.00365 | 1.00810 | 1.01119 | 1.00501 |
| Age | 1.03488 | 1.03581 | 1.03560 | 1.03489 |
| Gender | 1.02451 | 1.02442 | 1.02457 | 1.02470 |
| Hypertension | 1.01951 | 1.02001 | 1.02277 | 1.02063 |
| Diabetes | 1.01722 | 1.01755 | 1.01692 | 1.01688 |
| Coronary heart disease | 1.03178 | 1.03203 | 1.03225 | 1.03241 |
| TOAST | 1.01784 | 1.01778 | 1.01805 | 1.01781 |
| NIHSS | 1.45402 | 1.45664 | 1.45464 | 1.45251 |
| mRS | 1.44454 | 1.44473 | 1.44490 | 1.44503 |

AKD: acute kidney disease; sCr: serum creatinine; sCysC: serum cystatin C; eGFR: estimated glomerular filtration rate; TOAST: Trial of Org 10172 in Acute Stroke Treatment; NIHSS: National Institutes of Health Stroke Scale; mRS: modified Ranking Scale.

**Table S4. Analysis of the interaction between AKD and age in the Cox proportional hazard model**

|  | sCr based AKD | | | eGFR_sCr_ based AKD | | | eGFR_sCysC_ based AKD | | | eGFR_sCr+sCysC_ based AKD | | |
| --- | --- | --- | --- | --- | --- | --- | --- | --- | --- | --- | --- | --- |
|  | HR (95% CI) | P | P for interaction | HR (95% CI) | P | P for interaction | HR (95% CI) | P | P for interaction | HR (95% CI) | P | P for interaction |
| All-cause death |  |  | 0.59 |  |  | 0.62 |  |  | 0.38 |  |  | 0.52 |
| Age<60 | 6.02(1.29-28.13) | 0.02 |  | 3.90(0.85-18.00) | 0.08 |  | 1.96(0.42-9.05) | 0.39 |  | 3.86(0.83-17.96) | 0.08 |  |
| Age≥60 | 2.50(1.06-5.87) | 0.03 |  | 2.07(1.04-4.11) | 0.04 |  | 0.90(0.41-2.00) | 0.80 |  | 1.73(0.78-3.83) | 0.18 |  |
| Stroke recurrence |  |  | 0.17 |  |  | 0.39 |  |  | 0.98 |  |  | 0.07 |
| Age<60 | 2.28(0.91-5.75) | 0.08 |  | 1.89(0.81-4.40) | 0.14 |  | 0.93(0.37-2.34) | 0.88 |  | 1.80(0.77-4.21) | 0.18 |  |
| Age≥60 | 0.83(0.30-2.24) | 0.71 |  | 1.18(0.64-2.21) | 0.60 |  | 0.94(0.54-1.65) | 0.83 |  | 0.58(0.24-1.43) | 0.24 |  |

AKD: acute kidney disease; sCr: serum creatinine; sCysC: serum cystatin C; eGFR: estimated glomerular filtration rate; HR: hazard ratio; CI: confidence intervals.

**Table S5. Analysis of the interaction between AKD and gender in the Cox proportional hazard model**

|  | sCr based AKD | | | eGFR_sCr_ based AKD | | | eGFR_sCysC_ based AKD | | | eGFR_sCr+sCysC_ based AKD | | |
| --- | --- | --- | --- | --- | --- | --- | --- | --- | --- | --- | --- | --- |
|  | HR (95% CI) | P | P for interaction | HR (95% CI) | P | P for interaction | HR (95% CI) | P | P for interaction | HR (95% CI) | P | P for interaction |
| All-cause death |  |  | 0.62 |  |  | 0.48 |  |  | 0.62 |  |  | 0.50 |
| Male | 2.98(1.25-7.09) | 0.01 |  | 2.53(1.21-5.30) | 0.01 |  | 1.15(0.51-2.58) | 0.74 |  | 2.18(0.97-4.91) | 0.06 |  |
| Female | 1.93(0.44-8.48) | 0.38 |  | 1.43(0.42-4.93) | 0.57 |  | 0.71(0.17-3.07) | 0.65 |  | 1.13(0.26-4.92) | 0.87 |  |
| Stroke recurrence |  |  | 0.78 |  |  | 0.89 |  |  | 0.61 |  |  | 0.52 |
| Male | 1.34(0.59-3.06) | 0.48 |  | 1.36(0.73-2.53) | 0.34 |  | 0.86(0.47-1.57) | 0.62 |  | 0.77(0.34-1.76) | 0.54 |  |
| Female | 1.04(0.32-3.34) | 0.94 |  | 1.26(0.54-2.96) | 0.59 |  | 1.12(0.51-2.49) | 0.77 |  | 1.11(0.44-2.79) | 0.82 |  |

AKD: acute kidney disease; sCr: serum creatinine; sCysC: serum cystatin C; eGFR: estimated glomerular filtration rate; HR: hazard ratio; CI: confidence intervals.

**Table S6. Analysis of the interaction between AKD and diabetes in the Cox proportional hazard model**

|  | sCr based AKD | | | eGFR_sCr_ based AKD | | | eGFR_sCysC_ based AKD | | | eGFR_sCr+sCysC_ based AKD | | |
| --- | --- | --- | --- | --- | --- | --- | --- | --- | --- | --- | --- | --- |
|  | HR (95% CI) | P | P for interaction | HR (95% CI) | P | P for interaction | HR (95% CI) | P | P for interaction | HR (95% CI) | P | P for interaction |
| All-cause death |  |  | 0.94 |  |  | 0.34 |  |  | 0.09 |  |  | 0.15 |
| Non-diabetes | 2.84(1.01-7.97) | 0.05 |  | 2.76(1.27-6.00) | 0.01 |  | 0.54(0.17-1.76) | 0.31 |  | 1.08(0.33-3.50) | 0.90 |  |
| Diabetes | 2.48(0.84-7.33) | 0.10 |  | 1.42(0.48-4.17) | 0.52 |  | 1.82(0.72-4.62) | 0.20 |  | 2.94(1.17-7.43) | 0.02 |  |
| Stroke recurrence |  |  | 0.17 |  |  | 0.76 |  |  | 0.74 |  |  | 0.82 |
| Non-diabetes | 1.63(0.80-3.33) | 0.18 |  | 1.22(0.66-2.27) | 0.52 |  | 0.87(0.49-1.54) | 0.63 |  | 0.94(0.46-1.93) | 0.88 |  |
| Diabetes | 0.44(0.06-3.24) | 0.42 |  | 1.79(0.75-4.25) | 0.19 |  | 1.17(0.49-2.77) | 0.73 |  | 0.83(0.26-2.69) | 0.76 |  |

AKD: acute kidney disease; sCr: serum creatinine; sCysC: serum cystatin C; eGFR: estimated glomerular filtration rate; HR: hazard ratio; CI: confidence intervals.

**Table S7. Analysis of the interaction between AKD and hypertension in the Cox proportional hazard model**

|  | sCr based AKD | | | eGFR_sCr_ based AKD | | | eGFR_sCysC_ based AKD | | | eGFR_sCr+sCysC_ based AKD | | |
| --- | --- | --- | --- | --- | --- | --- | --- | --- | --- | --- | --- | --- |
|  | HR (95% CI) | P | P for interaction | HR (95% CI) | P | P for interaction | HR (95% CI) | P | P for interaction | HR (95% CI) | P | P for interaction |
| All-cause death |  |  | 0.80 |  |  | 0.58 |  |  | 0.40 |  |  | 0.32 |
| Non-hypertension | 2.44(0.56-10.71) | 0.24 |  | 2.98(0.99-8.98) | 0.05 |  | 0.49(0.07-3.62) | 0.48 |  | 0.77(0.10-5.78) | 0.80 |  |
| Hypertension | 2.82(1.19-6.71) | 0.02 |  | 1.94(0.90-4.18) | 0.09 |  | 1.16(0.54-2.51) | 0.70 |  | 2.23(1.03-4.81) | 0.04 |  |
| Stroke recurrence |  |  | 0.78 |  |  | 0.75 |  |  | 0.69 |  |  | 0.61 |
| Non-hypertension | 1.38(0.43-4.46) | 0.59 |  | 1.52(0.60-3.83) | 0.37 |  | 1.09(0.43-2.74) | 0.85 |  | 0.67(0.16-2.73) | 0.57 |  |
| Hypertension | 1.14(0.50-2.61) | 0.75 |  | 1.32(0.73-2.41) | 0.36 |  | 0.92(0.53-1.62) | 0.78 |  | 1.02(0.51-2.01) | 0.96 |  |

AKD: acute kidney disease; sCr: serum creatinine; sCysC: serum cystatin C; eGFR: estimated glomerular filtration rate; HR: hazard ratio; CI: confidence intervals.

**Table S8. Analysis of the interaction between AKD and NIHSS in the Cox proportional hazard model**

|  | sCr based AKD | | | eGFR_sCr_ based AKD | | | eGFR_sCysC_ based AKD | | | eGFR_sCr+sCysC_ based AKD | | |
| --- | --- | --- | --- | --- | --- | --- | --- | --- | --- | --- | --- | --- |
|  | HR (95% CI) | P | P for interaction | HR (95% CI) | P | P for interaction | HR (95% CI) | P | P for interaction | HR (95% CI) | P | P for interaction |
| All-cause death |  |  | 0.82 |  |  | 0.39 |  |  | 0.39 |  |  | 0.17 |
| NIHSS <5 | 2.03(0.48-8.55) | 0.34 |  | 1.66(0.50-5.50) | 0.41 |  | 0.33(0.04-2.41) | 0.27 |  | 1.07(0.25-4.54) | 0.92 |  |
| NIHSS 5-15 | 3.11(1.27-7.60) | 0.01 |  | 2.14(2.93-4.94) | 0.08 |  | 1.60(0.72-3.54) | 0.25 |  | 2.04(0.84-4.95) | 0.11 |  |
| NIHSS >15 | -- | -- |  | 3.54(0.35-35.31) | 0.28 |  | -- | -- |  | -- | -- |  |
| Stroke recurrence |  |  | 0.47 |  |  | 0.77 |  |  | 0.91 |  |  | 0.26 |
| NIHSS <5 | 0.78(0.25-2.45) | 0.67 |  | 1.22(0.59-2.51) | 0.59 |  | 0.87(0.44-1.72) | 0.69 |  | 0.56(0.21-1.53) | 0.26 |  |
| NIHSS 5-15 | 2.06(0.89-4.79) | 0.09 |  | 1.58(0.75-3.32) | 0.23 |  | 1.16(0.59-2.29) | 0.66 |  | 1.53(0.69-3.35) | 0.29 |  |
| NIHSS >15 | -- | -- |  | 1.96(0.18-21.06) | 0.58 |  | -- | -- |  | -- | -- |  |

AKD: acute kidney disease; sCr: serum creatinine; sCysC: serum cystatin C; eGFR: estimated glomerular filtration rate; HR: hazard ratio; CI: confidence intervals; NIHSS: National Institutes of Health Stroke Scale.

**Table S9. The goodness-of-fit analysis of multivariable model**

|  | sCr based AKD | | eGFRsCr based AKD | | eGFR_sCysC_ based AKD | | eGFR_sCr+sCysC_ based AKD | |
| --- | --- | --- | --- | --- | --- | --- | --- | --- |
|  | χ^2^ | P**^a^** | χ^2^ | P**^a^** | χ^2^ | P**^a^** | χ^2^ | P**^a^** |
| All-cause death | 10.41 | 0.24 | 8.90 | 0.35 | 11.22 | 0.19 | 12.06 | 0.15 |
| Stroke recurrence | 4.33 | 0.83 | 10.29 | 0.25 | 7.61 | 0.47 | 5.85 | 0.66 |

^a^ Accessed by Hosmer-Lemeshow test; AKD: acute kidney disease; sCr: serum creatinine; sCysC: serum cystatin C; eGFR: estimated glomerular filtration rate.
